# The Human Hookworm Vaccine

**DOI:** 10.1016/j.vaccine.2012.11.034

**Published:** 2013-04-18

**Authors:** Peter J. Hotez, David Diemert, Kristina M. Bacon, Coreen Beaumier, Jeffrey M. Bethony, Maria Elena Bottazzi, Simon Brooker, Artur Roberto Couto, Marcos da Silva Freire, Akira Homma, Bruce Y. Lee, Alex Loukas, Marva Loblack, Carlos Medicis Morel, Rodrigo Correa Oliveira, Philip K. Russell

**Affiliations:** a Sabin Vaccine Institute Product Development Partnership, Houston, TX, United States; b Sabin Vaccine Institute Product Development Partnership, Washington, DC, United States; c Sabin Vaccine Institute and Texas Children's Hospital Center for Vaccine Development, National School of Tropical Medicine, Baylor College of Medicine, Houston, TX, United States; d Department of Pediatrics and Molecular Virology and Microbiology, National School of Tropical Medicine, Baylor College of Medicine, Houston, TX, United States; e Department of Microbiology, Immunology, and Tropical Medicine, George Washington University School of Medicine and Health Sciences, Washington, DC, United States; f Public Health Computational and Operations Research (PHICOR), University of Pittsburgh School of Medicine, Pittsburgh, PA, United States; g Faculty of Infectious and Tropical Disease, London School of Hygiene and Tropical Medicine, UK; h Oswaldo Cruz Foundation (FIOCRUZ), Rio de Janeiro, Brazil; i Queensland Tropical Health Alliance, James Cook University, Cairns, Australia; j Oswaldo Cruz Foundation (FIOCRUZ) – René Rachou Research Centre, Belo Horizonte, Brazil

**Keywords:** Hookworm, Helminth, *Na*-GST-1, *Na*-APR-1, Anemia

## Abstract

Hookworm infection is one of the world's most common neglected tropical diseases and a leading cause of iron deficiency anemia in low- and middle-income countries. A Human Hookworm Vaccine is currently being developed by the Sabin Vaccine Institute and is in phase 1 clinical testing. The candidate vaccine is comprised of two recombinant antigens known as *Na*-GST-1 and *Na*-APR-1, each of which is an important parasite enzyme required for hookworms to successfully utilize host blood as a source of energy. The recombinant proteins are formulated on Alhydrogel^®^ and are being tested in combination with a synthetic Toll-like receptor 4 agonist. The aim of the vaccine is to induce anti-enzyme antibodies that will reduce both host blood loss and the number of hookworms attached to the gut. Transfer of the manufacturing technology to the Oswaldo Cruz Foundation (FIOCRUZ)/Bio-Manguinhos (a Brazilian public sector developing country vaccine manufacturer) is planned, with a clinical development plan that could lead to registration of the vaccine in Brazil. The vaccine would also need to be introduced in the poorest regions of Africa and Asia, where hookworm infection is highly endemic. Ultimately, the vaccine could become an essential tool for achieving hookworm control and elimination, a key target in the 2012 London Declaration on Neglected Tropical Diseases.

## Highlights

► Human hookworm infection is a leading cause of iron deficiency anemia. ► An estimated 700 million people in developing countries are affected. ► The Sabin Vaccine Institute PDP is developing the vaccine in collaboration with FIOCRUZ. ► The vaccine comprises two recombinant protein antigens on alum and a TLR4 agonist. ► The partnership's plan is that the vaccine will be licensed by 2020.

## 1 Background

Hookworm infection is a leading cause of iron-deficiency anemia in rural areas of the world's poorest countries [1]. An estimated 700 million people chronically harbor hookworms in their intestines—most of these people survive on less than $1–2 per day, a benchmark threshold for defining global poverty [1,2]. Indeed, hookworm infection is considered to be among the two most common chronic infections of the “bottom billion” and based on disability-adjusted life years (DALYs) lost, it is the most important neglected tropical disease (NTD) and the second most important parasitic infection (after malaria) [2,3].

Hookworms can live for years in the human intestine where they feed on host blood. Most of the morbidity is due to chronic blood loss that results in iron-deficiency anemia and hypoalbuminemia [1]. Recent evidence points to hookworm infection emerging as an important global threat to maternal–child health. Both children and pregnant women are especially vulnerable because of their higher iron demands and lower baseline iron reserves [4,5]. Children with moderate and heavy hookworm infections develop growth stunting and intellectual, cognitive, and educational deficits [4]. As they become adults entering the workforce, individuals with chronic hookworm infection experience substantial reductions in wage-earning potential [6]. Moreover between one-quarter and one-third of pregnant women in Africa are infected with hookworms, which can result in severe anemia, increased maternal morbidity and mortality, and fetal loss or prematurity [5]. Thus, hookworm infection is a major impediment to achieving Millennium Development Goals (MDGs) and targets for ameliorating poverty and improving maternal and child health [4].

Adding to the disease burden resulting from hookworm infection is the observation made in sub-Saharan Africa that there is extensive geographic overlap with malaria, and hookworm–malaria co-infections are widespread [7]. The effect of concurrent hookworm and malaria infections on the severity of anemia has been shown to be additive or synergistic, and malaria infections on the incidence of anemia have been shown to be synergistic and increase the risk of severe and profound anemia [8].

A Human Hookworm Vaccine is being developed by the product development partnership (PDP) of the Sabin Vaccine Institute [9,10]. The vaccine is being designed to target *Necator americanus*, the hookworm species responsible for approximately three-quarters or more of all human hookworm infections [11]. The eventual goal is to license a vaccine that contains two recombinant hookworm antigens, *Na*-GST-1 and *Na*-APR-1, which are formulated on an aluminum hydroxide adjuvant (Alhydrogel^®^). Clinical testing will evaluate whether an additional adjuvant, an aqueous formulation of a synthetic Toll-like receptor 4 (TLR 4) agonist (glucopyranosyl lipid A [GLA-AF]), will be required to achieve acceptable immunogenicity [9].

*Na*-GST-1 is a 24 kDa recombinant *N. americanus* glutathione-*S*-transferase expressed in yeast (*Pichia pastoris*) [12,13], while *Na*-APR-1 is a 45 kDa recombinant *N. americanus* aspartic protease expressed in tobacco plants. For safety and stability reasons, *Na*-APR-1 was modified through site-directed mutagenesis to produce a recombinant protein devoid of proteolytic activity [14,15]. Preclinical proof-of-concept that both recombinant *Na*-GST-1 and *Na*-APR-1 can induce protective efficacy has been demonstrated though challenge studies conducted in laboratory animals (reviewed in [9]).

• *Na*-GST-1 has been manufactured at pilot scale according to current Good Manufacturing Practices (cGMP). Following regulatory submissions in the United States and Brazil, *Na*-GST-1 is currently in phase 1 clinical trials in healthy adult volunteers in the United States and Brazil.
• *Na*-APR-1 has also undergone cGMP manufacture at pilot scale. An investigational new drug (IND) filing for this antigen formulated on Alhydrogel^®^ will be submitted to the United States Food and Drug Administration and the Brazilian regulatory agency (the Agencia Nacional de Vigilância em Saúde) in late 2012 or 2013.

Following phase 1 testing of each hookworm vaccine candidate antigen in adults and children, they will be combined into a single product, assuming that both have been shown to be safe and immunogenic. This co-formulated product will be tested in phase 2b and 3 studies in hookworm-endemic regions of Brazil and likely sub-Saharan Africa to evaluate its efficacy in preventing moderate and heavy infections and the resulting intestinal blood loss and anemia. In addition, early in clinical development a proof-of-concept challenge trial is being considered in hookworm-naïve adult volunteers who are vaccinated and then challenged with infective *N. americanus* larvae. Previous studies of experimental hookworm infection have demonstrated that it is feasible, safe, and reasonably well tolerated (depending on the dose of infectious larvae) [16–18]. This vaccination-challenge trial would be conducted after the initial phase 1 trials of each recombinant antigen in adults to provide an early indication of their potential efficacy.

The target product profile of the Human Hookworm Vaccine includes the following important features [9]:

1. The vaccine is intended for children under the age of 10 years who are at risk for acquiring moderate and heavy hookworm infections in endemic areas of developing countries.
2. The vaccine will be administered by intramuscular injection up to two doses and will require storage between 2 °C and 8 °C.
3. The vaccine can be administered concurrently with other childhood vaccines such as the measles vaccine.
4. Vaccine efficacy of at least 80% in preventing moderate and heavy hookworm infections caused by *N. americanus*.

Widespread use of an effective Human Hookworm Vaccine would significantly improve global public health and as outlined below could also become a critical technology for the eventual elimination of hookworm infection in low- and middle-income countries. Such a vaccine has been described as an ‘antipoverty vaccine’ because of its potential to improve the economic development of affected populations in addition to its positive impact on health [10]. In addition, due to the synergistic effect of concurrent infection with malaria and hookworm on incidence of anemia, using the vaccine in sub-Saharan Africa could potentially also reduce the burden of disease due to *Plasmodium falciparum*.

## 2 Main barriers and challenges

Licensure and global access to the Human Hookworm Vaccine will face significant scientific, programmatic, and commercial challenges, as described below.

### 2.1 Scientific challenges

Currently there are no licensed anthelminthic vaccines for humans. Two experimental schistosomiasis vaccines are undergoing early stage clinical testing in Brazil and sub-Saharan Africa [19,20], while the Human Hookworm Vaccine is the only vaccine in clinical development for hookworm infection. Hookworms are complex multicellular parasites, so that producing an efficacious vaccine against this helminth is in some respects an even more formidable challenge than producing vaccines against unicellular parasites such as those that cause malaria and other protozoan infections. The feasibility of producing an efficacious vaccine against hookworm has been reviewed previously and is based on past successes in developing an effective irradiated larval canine hookworm vaccine and in demonstrating the efficacy of recombinant protein vaccines in laboratory animal-challenge studies [9,21].

The major scientific hurdles in developing a hookworm vaccine for humans can be summarized as follows, and have been reviewed previously in Refs. [9,21]:

1. Achieving the target product profile for the Human Hookworm Vaccine will require that the vaccine induces high levels of antigen-specific antibodies. These antibodies will target two key parasite enzymes involved in both hemoglobin digestion and maturation of hookworm larvae into adult worms that are attached to the host intestine. *Na*-GST-1 is a heme-binding protein that is thought to be involved in detoxification of free heme by the parasite, whereas *Na*-APR-1 is a hemoglobinase required for hemoglobin digestion (reviewed in [9]). In laboratory animals, antibodies induced against these two enzymes result in diminished host intestinal blood loss and prevention of anemia following challenge infection (reviewed in [9]). However, the level of anti-enzyme antibodies required to achieve sufficient neutralization that will prevent establishment of infection has not yet been determined (i.e., an antibody-based correlate of protection has not been derived). Additionally, it is not yet known whether Alhydrogel^®^ and GLA-AF will be adequate as adjuvants for promoting the production of the desired amounts of anti-enzyme antibodies.
2. *N. americanus* induces robust but mostly ineffective immune responses in the human host. Moreover, *N. americanus* hookworms have evolved to strongly immunomodulate and down-regulate the host immune response to enable parasite survival in the host for months or even years [22]. In the absence of protective immunity during natural infection, we have few clear leads to best direct the human immune system to reduce the number of hookworms in the gut and thereby reduce host blood loss.
3. To compound the problem outlined above, hookworm infection steers the immune response to parasites antigens (and possibly bystander antigens) toward strong Th2 responses, associated with increased levels of total and specific IgE antibodies [22–25]. This tendency to induce IgE to hookworm antigens has been shown to be especially true for those expressed by infective larvae after they penetrate the host skin, such as *Na*-ASP-2 [23]. Such antigens have been found to be unsuitable for clinical development, since when used as vaccine components they can induce immediate-type allergic reactions in previously exposed individuals [23]. As a result of this safety concern with larval antigens, PDP efforts are instead now focused on developing adult hookworm antigens, as described above [9].
4. Neither recombinant hookworm antigen induces sterilizing immunity in laboratory animals, nor is it expected that a hookworm vaccine will induce sterilizing immunity in humans. Instead it is anticipated that the Human Hookworm Vaccine will result in substantial reductions both in the number of hookworms and in the amount of hookworm-induced intestinal blood loss. Thus, the vaccine will prevent anemia and disease-producing moderate and heavy hookworm infections, but not necessarily subclinical light hookworm infections (as determined on the basis of quantitative fecal egg counts).
5. The minimally acceptable duration of protection for the Human Hookworm Vaccine has not been established. A recent analysis indicates that the vaccine must sustain protective immunity for at least 5 years to be cost-effective (see below).

### 2.2 Programmatic challenges

A key programmatic challenge to operationalizing an eventual licensed Human Hookworm Vaccine is to successfully incorporate it into existing control programs. Currently there are two products already in use, namely two anthelminthic drugs of the benzimidazole class (i.e., albendazole and mebendazole). In hookworm-endemic regions, each drug is currently being deployed in programs of regular ‘deworming’ or mass drug administration (MDA) in order to reduce the burden of hookworm and other soil-transmitted helminth infections such as ascariasis and trichuriasis. A 2001 World Health Assembly resolution called for the expansion of these deworming programs in order to reach annually (or twice or three-times annually in areas of high transmission) most or all of the world's school-aged children at risk for acquiring soil-transmitted helminth infections [26]. Today, these two drugs are largely being donated by GlaxoSmithKline and Johnson & Johnson for albendazole and mebendazole, respectively [26].

In sub-Saharan Africa and elsewhere in developing countries, hookworm infection is co-endemic not only with other soil-transmitted helminth infections, but also with several other NTDs, including schistosomiasis, lymphatic filariasis, onchocerciasis, and trachoma [4,27]. The January 2012 London Declaration on the NTDs emphasized the importance of targeting hookworm through increased control efforts, integrated with the control or elimination of several other NTDs [28]. The major emphasis of such efforts is based on MDA using a so-called “rapid impact package” of medicines donated by the pharmaceutical companies, which includes either albendazole or mebendazole, but also ivermectin, praziquantel, and azithromycin [4,26,27].

Whereas MDA for NTDs such as lymphatic filariasis, onchocerciasis, and trachoma has been shown to result in the elimination of these diseases in more than two dozen countries, to date it has not resulted in the elimination of hookworm or the other soil-transmitted helminths [29]. The reasons for this failure are several:

1. According to a recent systematic review, mebendazole cures on average only 15% of hookworm infections, although reported egg count reduction rates (a surrogate measure of reduction in worm burden) are quite variable, ranging from 0% to as high as 68% for *N. americanus* infection, and 98% reduction for mixed *N. americanus* and *Ancylostoma duodenale* hookworm infections [30]. Similarly, mebendazole has not been shown to improve anemia prevalence when used as part of MDA [5]. The reasons for such drug failures are unclear, but the observation that repeated use of mebendazole in the same geographic area is associated with diminishing efficacy [31] has led some investigators to suggest the possibility of emerging drug resistance, although whether resistance has actually occurred is considered controversial and is as yet unproven [9].
2. Albendazole currently has a higher reported rate of cure for hookworm [30], but drug failure has also been reported [32] and in some areas of Africa post-treatment reinfection can occur in less than a year [33]. Rapid post-treatment reinfection in areas of high transmission should prompt twice or thrice annual treatment as recommended by the WHO [34]. However, such frequent deworming is often considered impractical or not feasible for logistical and cost reasons.
3. Deworming is carried out primarily in school-aged or preschool children. Whereas for ascariasis and trichuriasis the highest intensity (worm burden) infections occur in children, for hookworm infection it is typically the adults (including pregnant women) who have the highest worm burdens [35]. Therefore, whereas frequent and periodic deworming has been shown to interrupt transmission of *Ascaris lumbricoides* in endemic communities [36], hookworm transmission would be expected to continue unabated if only children are targeted with MDA. Such observations point to the need for universal coverage, i.e., deworming of children and adults, if hookworm elimination was to be targeted through use of MDA alone.

Global concerns about the effectiveness and/or sustainability of MDA for control of hookworm have prompted international efforts to develop and test technologies, which could complement deworming and possibly lead to the eventual elimination of hookworm infection as a cost-effective public health control measure.

**Fig. 1:**
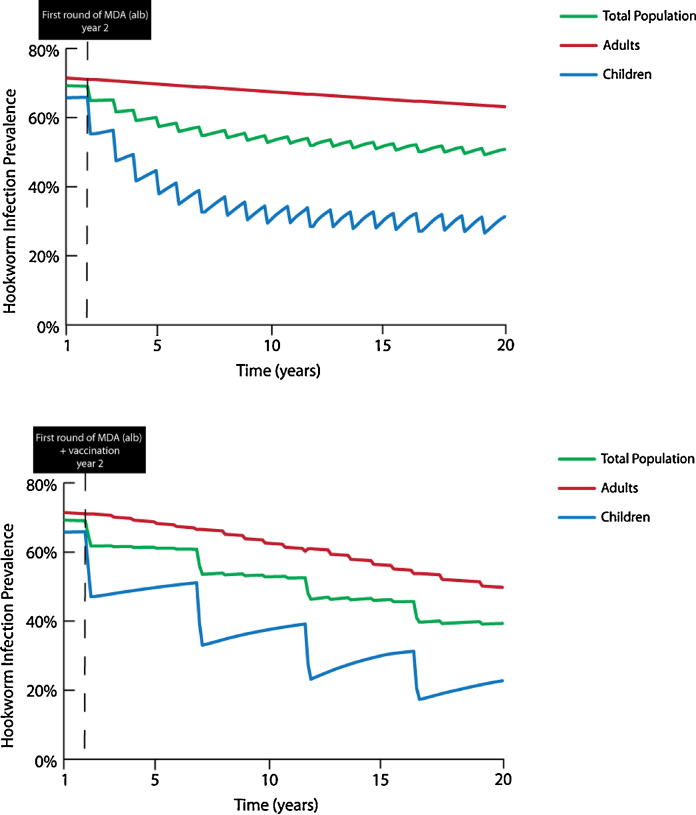
Benefit of adding an effective Human Hookworm Vaccine to Mass Drug Administration (MDA) in order to achieve hookworm elimination, using an economic dynamic transmission compartment model with compartments representing human and free-living hookworm populations. In this scenario, albendazole is administered annually to 75% of children ages 1–14 and the MDA + vaccination (2 doses) is administered to the same group once every 5 years (assuming a 5-year duration of vaccine protection). Albendazole cure rate = 78.4%; vaccine efficacy = 70%; mean baseline worm burden = 30 (adults), 15 (children). Top: MDA alone. Bottom: MDA + vaccination.

**Fig. 2:**
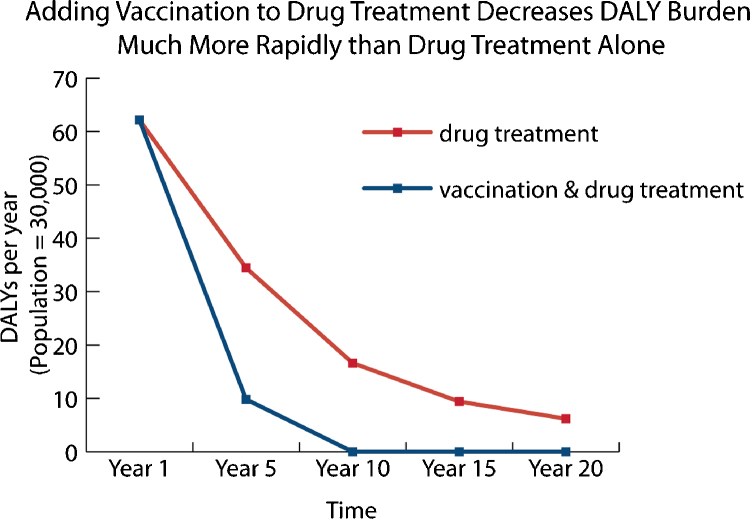
Benefit of an effective Human Hookworm Vaccine in reducing disease burden, as measured by disability adjusted life years (DALYs), relative to MDA alone. Only disability resulting from anemia is included in this analysis. Albendazole cure rate = 78.4%; vaccine efficacy = 70%; mean baseline worm burden = 30 (adults), 15 (children).

An independent modeling exercise has concluded that compared with regular MDA, an effective hookworm vaccine would be cost-effective (in many cases, highly cost-effective or even cost-saving, that is ‘economically dominant’) across a large number of scenarios of vaccine cost and prevalence of infection [37 and unpublished results]. In this analysis, when combined with MDA a hookworm vaccine led to cost savings and improved health compared to MDA alone for both school-aged children and non-pregnant women of reproductive age as long as the vaccine was at least 30% effective in preventing infection, 40% effective in reducing egg production, and cost less than $100 per fully vaccinated individual [37]. Additional analyses have indicated that a vaccine that induces protection of at least 5 years’ duration could lead to the interruption of hookworm transmission and reduce the burden of disease among both children and adults [unpublished results] (Fig. 1), which is a necessary requirement for the elimination of hookworm in endemic areas [29]. These scenarios include the possibility of vaccinating children following administration of an anthelminthic (‘vaccine-linked chemotherapy’), which would result in a significantly enhanced and more rapid reduction in disease burden (as measured by DALYs) relative to MDA alone [unpublished results] (Fig. 2).

Since it is impossible to predict if pre-vaccination administration of an anthelminthic or continued exposure following vaccination will have an effect (either positive or negative) on vaccine-induced immunity, the effect of these two potential modifiers was assumed to be neutral in the modeling exercises mentioned above. Questions such as these will be investigated during clinical trials of the vaccine. In addition, since the number of vaccine doses that will be needed to induce protective immunity in a vaccinated individual is currently unknown, it has been assumed to be two for the purposes of planning and modeling.

Despite the assumed economic dominance of the Human Hookworm Vaccine, its comparative cost effectiveness relative to deworming, and the vaccine's potential for interrupting transmission and effecting elimination, the idea of a vaccine-centered approach to hookworm control has not been widely discussed or debated by the global public health community. There are several possible reasons for this, including:

1. The public health community committed to the control and elimination of the NTDs is comprised predominantly of experts in MDA. Because no NTD vaccines have yet been licensed there is little or no familiarity with how such products might be incorporated into existing control programs.
2. Current public health efforts have focused on the concept of *control* of soil-transmitted helminths rather than their elimination [28,29]. As outlined above a full consideration of helminth transmission dynamics strongly suggests that MDA is not sufficient and that a vaccine will be required to eliminate hookworm [29], although MDA may be sufficient to eliminate ascariasis and trichuriasis [36].
3. Anthelminthic vaccines are still in early stages of clinical development and are several years from being licensed.

These perceptions and attitudes represent barriers to the widespread introduction and uptake of a newly licensed vaccine for hookworm. To counter these barriers, a demand forecast for the Human Hookworm Vaccine is now being conducted under the auspices of the Bill & Melinda Gates Foundation, which will assess the potential end-users of the vaccine and help the Sabin Vaccine Institute to develop a roadmap to eventual global use of the vaccine in endemic regions.

### 2.3 Commercial challenges

Similar to other major NTDs, hookworm infection occurs almost exclusively among the poorest people living in low- and middle-income countries [3]. Given that almost three-quarters of a billion people are currently infected with hookworm, the potential market for a Human Hookworm Vaccine is vast. However, there is little if any *commercial* potential for such a product given that it would be used exclusively for the benefit of the world's poorest. This lack of financial incentive contrasts with that of vaccines for HIV/AIDS or tuberculosis, which is endemic not only in developing countries but also in North America, Europe, and in some of the wealthier Asian nations. To overcome this commercial barrier to development and eventual use of a licensed hookworm vaccine, the Sabin Vaccine Institute is partnering with public sector vaccine manufacturers in so-called innovative developing countries (IDCs) such as Brazil and Mexico [38]. These organizations belong to the Developing Country Vaccine Manufacturers Network (DCVMN), which shares information about best practices for manufacture, regulatory affairs, and vaccine introduction [39].

For the Human Hookworm Vaccine, the Sabin Vaccine Institute is collaborating with the Oswaldo Cruz Foundation (FIOCRUZ) for both clinical testing and industrial-scale manufacture [10]. To improve and scale up the processes for producing vaccine supplies suitable for phase 2 and phase 3 clinical trials, and ultimately for the industrial-scale manufacture of the vaccine, efforts have been initiated to transfer the manufacturing technology for both *Na*-GST-1 and *Na*-APR-1 to FIOCRUZ, specifically with its vaccine production division known as Bio-Manguinhos. Ultimately, FIOCRUZ/Bio-Manguinhos will be a major global producer of the Human Hookworm Vaccine, using an unrestricted license from the Sabin Vaccine Institute. Preliminary estimates indicate that a vaccine consisting of the two recombinant proteins (manufactured using the current methodologies) and adjuvanted with Alhydrogel^®^ could be produced for less than $1 per dose. The current regulatory strategy is to pursue registration in Brazil in parallel with an application to the WHO for prequalification of the Brazilian-produced product, with distribution of vaccine to endemic countries worldwide through international procurement agencies such as UNICEF and the Pan-American Health Organization's Revolving Fund.

## 3 Future perspectives and concrete actions

Several concrete actions will be taken in the coming years to advance the development of the Human Hookworm Vaccine, which if successful will ultimately lead to the vaccine's licensure by 2020:

1. *Clinical development*. Following phase 1 studies of each of the recombinant antigens that will be included in the bivalent vaccine, they will be tested in combination for their ability to prevent infection and intestinal blood loss. Such studies will be conducted in children through a “proof-of-concept” phase 2b study in Brazil in collaboration with the Rene Rachou Research Center in Minas Gerais State (a member institute of the FIOCRUZ network). In addition, the Sabin Vaccine Institute is developing plans to provide an early assessment of efficacy by vaccinating adult volunteers in the United States and challenging them with *N. americanus* infectious larvae.
2. *Product development*. In parallel, the Sabin Vaccine Institute has initiated technology transfer to FIOCRUZ/Bio-Manguinhos in order to produce vaccine supplies for phase 2 and phase 3 testing. Goals of the manufacturing technology transfer include improvement of protein expression yields for both *Na*-GST-1 and *Na*-APR-1, and to manufacture a co-formulated product containing both antigens.
3. *Regulatory strategy*. The Sabin Vaccine Institute, in partnership with FIOCRUZ, intends to first register the vaccine in Brazil while in parallel applying to the WHO for prequalification. However, other options for regulatory approval are also under consideration.
4. *Demand forecasting*. This exercise will be extremely important in providing justification for continued vaccine development and will be essential to developing a roadmap to vaccine introduction and uptake in Brazil and elsewhere in Latin America, as well as in areas of high hookworm transmission in Africa and Asia. A critical component of demand forecasting will require detailed estimates of the costs for manufacturing and distributing the vaccine.

## 4 Conclusions and lessons learned

Hookworm infection is one of the most common infections of the world's poorest people. The Human Hookworm Vaccine is a key technology for the Global Vaccine Action Plan and the Decade of Vaccines and an essential tool for achieving the MDGs, especially those linked to maternal and child health. Product and clinical development plans, as well as regulatory and global access strategies, are in place for this vaccine, with the recognition that development and vaccine introduction face important scientific, programmatic, and commercial challenges as outlined above.

Among the lessons learned over the last decade of international cooperation between the Sabin Vaccine Institute and FIOCRUZ are:

1. The importance of a strong evidence base to guide and justify vaccine development. The scientists associated with the Sabin Vaccine Institute have published more than 100 papers on hookworm vaccine development in the peer-reviewed biomedical literature.
2. Frequent scientific exchanges and tight project and program management are important to ensure success in technology transfer from the Sabin Vaccine Institute product development laboratories to cGMP manufacturers (especially in IDC nations), as are regular program reviews to assess the feasibility, integrity and reproducibility of the manufacturing processes.
3. Developing a regulatory strategy and clinical development plans in collaboration with regulatory agencies in the United States and Brazil is critical to maintaining timelines and ultimately licensing a vaccine.
4. The importance of advocacy to make the public health and scientific community aware of the enormous threat of hookworm infection to the health of at-risk children and pregnant women living in low- and middle-income countries, together with a program of education to inform the global public health community about the limitations of MDA as a single-dimension strategy for the control and eventual elimination of hookworm infection.

In summary, the Human Hookworm Vaccine is an important new technology and one that has the potential to significantly reduce the prevalence and burden of iron-deficiency anemia in low- and middle-income countries, to help achieve MDG targets related to maternal and child health, and to help reduce poverty in the poorest regions of Africa, Asia, and Latin America. The Human Hookworm Vaccine Initiative will also prove instructive for existing and new potential vaccine development initiatives targeting neglected tropical diseases.

## Conflict of interest statement

All authors serve in various roles in the product development partnership described in the manuscript and are involved in different aspects of the development of a vaccine against hookworm as described. Several of the authors are inventors on patents related to the different hookworm vaccine antigens.
